# The impact of antenatal care utilization on admissions to neonatal intensive care units and perinatal mortality in Georgia

**DOI:** 10.1371/journal.pone.0242991

**Published:** 2020-12-02

**Authors:** Tinatin Manjavidze, Charlotta Rylander, Finn Egil Skjeldestad, Nata Kazakhashvili, Erik Eik Anda

**Affiliations:** 1 Department of Community Medicine, Faculty of Health Sciences, University of Tromsø –The Arctic University of Norway, Tromsø, Norway; 2 Department of Public Health, Faculty of Medicine, Ivane Javakhishvili Tbilisi State University, Tbilisi, Georgia; African Population and Health Research Center, KENYA

## Abstract

**Introduction:**

Appropriate antenatal care (ANC) utilization has direct, significant effects on perinatal mortality (PM). Georgia has one of the highest PM rates (11.7 per 1000 births) in Europe and launched a more intensive ANC programme in 2018.

**Aim:**

To evaluate the associations between the Adequacy of Prenatal Care Utilization (APNCU) index and neonatal intensive care unit (NICU) admission and PM in Georgia.

**Methods:**

The Georgian Birth Registry (GBR), with linkage to the Vital Registration System, was used as the main data source; 148,407 eligible mothers and singleton newborns were identified during the observation period (2017–2019). The main exposure was ANC utilization, measured by the APNCU index, and the hospitalization registry was used to validate NICU admissions. Logistic regression analysis was used to assess the associations between the exposure and outcomes while controlling for potential confounders.

**Results:**

The overall PM rate was 11.6/1000 births, and the proportion of newborns with a NICU admission was 7.8%. 85% of women initiated ANC before gestational age week 12. According to the APNCU index, 16% of women received inadequate, 10% intermediate, 38% adequate, and 36% intensive care. Women who received intermediate care had the lowest odds of PM (adjusted odds ratio [AOR] = 0.56, 95% confidence interval [CI] 0.45–0.70), and newborns of women who received inadequate care had the highest odds of NICU admission (AOR = 1.16, 95% CI 1.09–1.23) and PM (AOR = 1.18, 95% CI 1.02–1.36).

**Conclusion:**

ANC utilization is significantly associated with newborn asmissions to NICU and PM in Georgia. Women received inadequate care experienced the highest odds of newborn admissions to NICU and PM.

## Introduction

Interventions before and during labour may reduce the risk of perinatal mortality (PM). Timely initiation of antenatal care (ANC) and attending ANC as recommended are the major strategic interventions available to improve newborn health outcomes. PM is strongly associated with maternal conditions like gestational diabetes, anaemia, hypertensive disorders, preterm labour, and intrauterine growth restriction [1], all of which can be identified and managed through proper ANC. Approximately 33% of stillbirths and 71% of neonatal deaths can be avoided if the coverage and quality of preconception care, ANC, and intrapartum care are improved [2]. Previous research has suggested that differences in PM across countries in the European Union could be explained by differences in the quality of ANC [3].

To be considered high quality, ANC must be initiated before gestational age (GA) week 12, and subsequent visits must be attended at recommended intervals throughout the pregnancy. In 2013, 58.6% of pregnant women globally attended ANC before GA week 12, but there were large regional variations [4]. Maternal education, socioeconomic status, healthcare access, family support, and previous pregnancy experience are recognized as major determinants of timely initiation of ANC. Less than three ANC visits was associated with increased risks of PM in Australia [5]. A study from the USA showed that the risk of preterm birth, stillbirth, early and late neonatal mortality increased as the number of ANC visits decreased [6].

Newborn admission to a neonatal intensive care unit (NICU) is another significant indicator of perinatal health. NICU admission and PM are related outcomes, since newborns are admitted to the NICU for severe medical conditions. More advanced medical technologies and better resource utilization may explain increases in newborn survival [7, 8]. Studies of women with low-risk pregnancies showed that, in limited-resource settings, reduced ANC visits did not have any effect on NICU admissions, but they were associated with an increase in PM [9]. Additionally, the risk of mortality or severe morbidity, which results in NICU admission, was two times higher for extremely preterm newborns whose mothers did not receive optimal ANC compared to those whose mothers did [10]. The delivery of high-quality ANC to women with high-risk pregnancies, including risk assessment and treatment with corticosteroids and magnesium sulphate, may reduce the risk of extremely preterm birth and NICU admission [11–13].

The adequacy of ANC utilization can be measured by several different indices, which take into account GA week at ANC initiation and the number of ANC visits adjusted for GA [14–17]. However, studies on ANC utilization suggest that results differ significantly by the indices used [17, 18]. For years, the Kessner index has been widely used to assess the association between ANC and birth outcomes [19, 20]. At the end of the 1990s, the Adequacy of Prenatal Care Utilization (APNCU) index was proposed as a better way of measuring ANC utilization [16]. The APNCU index is based on GA week at ANC initiation and the subsequent number of ANC visits. Studies using the APNCU index have identified associations between ANC utilization and small-for-GA newborns, preterm birth, and infant mortality [6, 21].

The recommendations for providing high-quality ANC have changed over time. In 2002, the World Health Organization (WHO) recommended goal-oriented, focused ANC: four ANC visits during pregnancy, with the first visit taking place before GA week 12. In 2016, the WHO changed its recommendation to eight ANC visits, of which six should take place in the third trimester, citing that as a better strategy for improving maternal, foetal, and neonatal outcomes [9, 22].

Georgia is an upper-middle income country with a population of 3.72 million. In 2018, the PM rate in Georgia was 11.7 per 1000 births [23], which is one of the highest among European countries. Additionally, a high proportion of women in Georgia attend at least four ANC visits during pregnancy (80.8%) [23], based on aggregated data from medical facilities. The proportion of women attending four ANC visits and initiating ANC before GA week 12 is increasing; however, these numbers are still far from Georgia’s target of 100% by 2030 [24]. Our previous study showed that women who did not attend any ANC visits during pregnancy had two-fold higher odds of PM compared to women who attended at least one ANC visit [25].

To our knowledge, little is known about the relationship between adequacy of ANC utilization and NICU admission and PM, especially in countries with a relatively high PM rate. Additionally, the perinatal outcomes of different ANC groups remain uninvestigated in Georgia. The aims of this study were to evaluate the associations between the APNCU index and NICU admission and PM in Georgia.

## Materials and methods

### Study population

The Georgian Birth Registry (GBR) ensures registration of all medical facility-based deliveries and ANC visits. The coverage of newborns registered in the GBR is 99.8%; the remaining 0.2% are registration disparities between the GBR, civil registration, and home deliveries (which account for around 0.1% of all deliveries in Georgia) [23]. To supplement and validate GBR data, we used the Vital Registration System (VRS). The VRS registers all births and deaths in the country and is routinely used for GBR data validation. Without registering birth or death in VRS is it impossible to issue birth or death certificate, thus, a newborn cannot have a personal identification number and diseased person cannot be buried. It means that all births and death in the country (except very minor exceptions) are registered in the VRS. Outcomes for transferred or discharged newborns are not sufficiently registered in the GBR, thus VRS data is used to identify cases of early neonatal death (END) (including time of birth and death).

From January 1^st^ 2017 to December 31^st^ 2019, 152,798 newborns and 150,593 mothers were registered in the GBR. After merging GBR and VRS data, we identified 1396 stillbirths (GBR) and 537 ENDs (VRS). We excluded 41 cases of ENDs which had a missing newborn and/or maternal ID. We also excluded multiple births (n = 2163), due to the higher risk of complications during pregnancy and delivery compared to singleton births, as well as women with parity >15 (n = 40), GA >43 weeks (n = 18), and maternal age >53 years (n = 7). Thus, 98.6% of women who delivered in medical facilities in Georgia during the study period met the inclusion criteria, for a final analytical sample of 148,407 singleton newborns and their mothers.

On February 1^st^ 2018, Georgia launched a new ANC programme, which is based on the latest WHO recommendations (i.e., eight ANC visits, of which six should be in the third trimester). Therefore, women were considered part of the ‘old’ ANC programme if they had any registered ANC visits before February 1^st^ 2018, or were beyond GA week 12 at February 1^st^ and initiated ANC thereafter. Women were considered part of the ‘new’ ANC programme if they had a registered first visit in the GBR after February 1^st^ 2018 and were below GA week 12 at February 1^st^. GA week 12 is the threshold at which women should initiate ANC to get financing from the state programme in Georgia.

### Exposure

The main exposure was defined as the utilization of antenatal care, as measured by the APNCU index [16]. This index characterizes ANC using two dimensions: GA week at ANC initiation and number of ANC visits, adjusted for GA week at ANC initiation and at GA week at delivery. For each GA week at delivery, the observed number of ANC visits is divided by the expected number of visits at that particular GA week. The APNCU index stratifies ANC into four categories: intensive care, adequate care, intermediate care, and inadequate care. We calculated the expected number of ANC visits according to the APNCU index based on the Georgian guidelines for the old and the new ANC programme separately (Table 1). The observed number of ANC visits was extracted from the GBR for each GA week at delivery. Women who had initiated ANC up to completed GA week 14 and attended 110% or more of recommended ANC visits were categorized as receiving intensive care. Women who had initiated ANC up to completed GA week 14 and attended 80%-109% of recommended ANC visits were classified as receiving adequate care (reference category). Women who had initiated ANC up to completed GA week 14 and attended 50%-79% of recommended ANC visits were categorized as receiving intermediate care, and those who had initiated ANC after completed GA week 14, attended less than 50% of recommended ANC visits, or did not have any ANC visits during pregnancy, were classified as receiving inadequate care.

**Table 1:** Number of expected antenatal care (ANC) visits for each gestational age (GA) week at delivery.

	Number of ANC visits–Before February 1^st^ 2018 (old ANC programme)			
**GA week**	Intensive care	Adequate care	Intermediate care	Inadequate care
≥37	≥6	4–5	2–3	0–1
32–36	≥5	3–4	1–2	0
22–31	≥4	2–3	1	0
	Number of ANC visits–After February 1^st^ 2018 (new ANC program)			
**GA week**	Intensive care	Adequate care	Intermediate care	Inadequate care
≥40	≥9	7–8	4–6	0–3
38–39	≥8	6–7	3–5	0–2
36–37	≥7	5–6	3–4	0–2
34–35	≥6	4–5	2–3	0–1
30–33	≥4	3	2	0–1
26–29	≥4	3	1–2	0
22–25	≥4	2–3	1	0

### Outcomes

Study outcomes were NICU admissions and PM. NICU admissions in the GBR were validated using the hospitalization registry, which ensures registration of all patients admitted to hospital, although there is no specific information about NICU admission. Of the 15,072 (10%) newborns with missing information on NICU admission in the GBR, 41 were found in the hospitalization registry during the early neonatal period. Thirty-five of these were categorized as having a NICU admission based on their condition, the urgency of the situation, and the organizational level of the hospital (only level 3 hospitals have a NICU in Georgia). The remaining six cases were classified as not having a NICU admission. In line with internationally accepted definitions, Georgian guidelines define stillbirth as the delivery of a newborn with no sign of life after completed GA week 22 and END as the death of a livebirth during the first 7 days (168 hours) of life. The combination of stillbirth and ENDs is defined as PM. Information on PM cases was validated using VRS data.

### Covariates

We drew a directed acyclic graph (DAG) to identify confounding factors. Based on the DAG, we adjusted our analysis for maternal age (≤19, 20–29, 30–39, ≥40 years), parity (nulliparous, multiparous), education (primary, secondary, higher), and region of residence and delivery (resided and delivered in Tbilisi, resided in Tbilisi and delivered outside Tbilisi, resided outside Tbilisi and delivered in Tbilisi, resided and delivered outside Tbilisi). GA was accounted for through the APNCU index.

### Statistical analyses

We calculated descriptive statistics for selected maternal characteristics across APNCU index categories. We used logistic regression analysis to assess the associations between APNCU index categories and NICU admission and PM, and conducted sensitivity analyses by running the same regression models stratified by: 1) the old/new ANC programme; and 2) preterm/term newborns. The results are presented as unadjusted odds ratios (ORs) and adjusted ORs (AORs) with 95% confidence intervals (CIs). The chi-square test was used to test whether there was a difference in the proportion of NICU admissions and PM before and after the implementation of the new ANC programme.

The APNCU index only evaluates ANC initiation before completed GA week 14. To enhance our understanding about the importance of timely ANC initiation, women were categorized into three groups: ANC initiation up to completed GA week 12 (reference category), ANC initiation after completed GA week 12 and before GA week 28, and ANC initiation in or after completed GA week 28. We then calculated the odds of PM and NICU admission for women with ANC initiation after completed GA week 12. Completed GA week 12 was used as a cut-off value, as the WHO has identified this as the best period in which to initiate ANC (22). Statistical software STATA (StataCorp, College Station, TX, USA) 16.0 version was used for the analysis.

### Ethical consideration

The NCDC Institutional Review Board revised and approved the study protocol (IRB # 2017–010 31.03.2017). The Regional Committee for Medical and Health Research Ethics, North Norway, approved the use of the data from the GBR for research purposes (2017/404/REK Nord). The data used for the research were fully anonymised and meet the criterion for privacy protection under the General Data Protection Regulation (GDPR).

## Results

**Fig 1:**
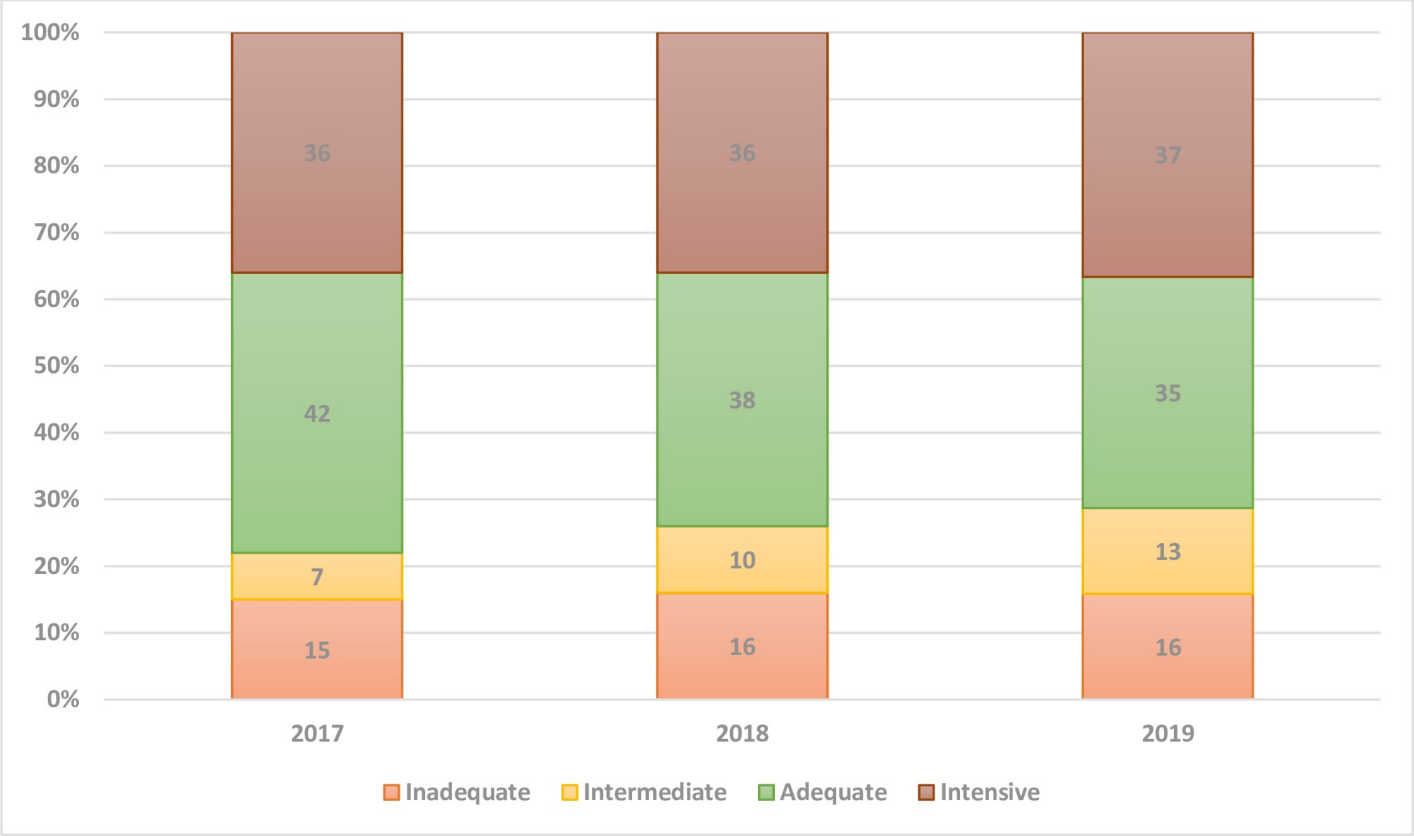
Adequacy of Prenatal Care Utilization index categories by study year (2017–2019) in mothers with a singleton pregnancy (%).

In total, 5.3% of our study women did not have any ANC visits registered in the GBR. According to the APNCU index, 16% of women received inadequate care, 10% intermediate care, 38% adequate care, and 36% intensive care. From 2017 to 2019, the proportion of women who received adequate care decreased, whereas the proportion receiving intermediate care increased slightly (Fig 1).

Mothers below 19 years of age had the highest proportion of inadequate care (23%) compared to mothers in other age groups. Seventeen percent of multiparous women and 26% of women with primary education received inadequate care, which was higher than the proportion of primiparous women with higher education. Women in the age group 30–39 years (38%) who gave birth to their first baby (41%) and had higher education (44%), had a higher proportion of intensive care than the other age groups, multiparous women, and women with primary education. Also, 42% of those who lived and delivered in Tbilisi received intensive care (Table 2).

**Table 2:** Maternal characteristics by Adequacy of Prenatal Care Utilization index categories presented as frequencies and row percentages.

	Intensive		Adequate		Intermediate		Inadequate	
	(n = 53,794), 36%		(n = 56,841), 38%		(n = 14,800), 10%		(n = 22,972), 16%	
Maternal age, years								
*≤19*	1047	28	1375	37	443	12	874	23
*20–29*	31,165	36	34,026	39	8814	10	12,948	15
*30–39*	19,693	38	19,547	37	5070	10	8109	16
*≥40*	1889	36	1893	36	473	10	1041	20
Parity								
*Nulliparous*	23,693	41	21,138	37	5182	9	7194	13
*Multiparous*	30,047	33	35,663	39	9608	11	15,756	17
Education								
*Primary*	2640	21	5143	42	1402	11	3135	26
*Secondary*	21,830	33	28,167	42	6548	10	10,370	16
*Higher*	23,280	44	19,169	36	5328	10	5453	10
*Unknown*	6044	38	4362	27	1522	10	4014	25
Region of residence/delivery								
*Lived and delivered in Tbilisi*	20,507	42	16,234	33	5397	11	7241	15
*Lived in Tbilisi*, *delivered outside Tbilisi*	440	34	467	36	185	14	195	15
*Lived outside Tbilisi*, *delivered in Tbilisi*	7721	36	7541	35	2558	12	3546	17
*Lived and delivered outside Tbilisi*	25,125	33	32,594	43	6655	9	11,878	16
Preterm birth	4500	8.4	3642	6.4	678	4.6	1995	8.7

Among the 148,407 singleton newborns, the NICU admission rate was 7.8% (95% CI 7.7–7.9); there were 1717 PM cases, 431 ENDs, and 1286 stillbirths. During the study period (2017–2019), the overall PM rate among singletons was 11.6 (95% CI 11.0–12.1) per 1000 births; END and stillbirth rates were 2.9 (95% CI 2.7–3.2) per 1000 livebirths and 8.7 (95% CI 8.2–9.1) per 1000 births, respectively. Women who received intermediate care had the lowest rates of NICU admission (7.0%), preterm birth (4.8%), PM (6.9 per 1000 births), ENDs (2.1 per 1000 livebirths), and stillbirth (4.8 per 1000 births). Rates of NICU admission (8.8%) and preterm birth (8.6%), as well as PM (16.9 per 1000 births), END (3.9 per 1000 livebirths), and stillbirth (13.1 per 1000 births) rates were higher among women who received inadequate care compared to the other groups (Tables 2 and 3).

**Table 3:** Neonatal intensive care unit (NICU) admission rates, perinatal mortality (PM), early neonatal death (END), and stillbirth (SB) by Adequacy of Prenatal Care Utilization index categories among singleton newborns.

	Intensive	Adequate	Intermediate	Inadequate
	(n = 53,794), 36%	(n = 56,841), 38%	(n = 14,800), 10%	(n = 22,972), 16%
NICU (%)	4469 (8.3)	4066 (7.2)	1035 (7.0)	2019 (8.8)
PM, n *(per 1000 births)*	561 (10.4)	665 (11.7)	102 (6.9)	389 (16.9)
ENDs, n *(per 1000 livebirths)*	161 (3.0)	151 (2.7)	31 (2.1)	88 (3.9)
SB, n *(per 1000 births)*	400 (7.4)	514 (9.0)	71 (4.8)	301 (13.1)

There were 82,330 mothers and singleton newborns in the old ANC programme, and 66,077 in the new ANC programme. The proportion of NICU admissions in the old and new ANC programmes was 6.8% (95% CI 6.6–7.0) and 9.1% (95% CI 8.8–9.3), respectively (*p*<0.01). The number of PM cases was 911 (261 ENDs and 650 stillbirths) and 806 (170 ENDs and 636 stillbirths), respectively. The proportion of PM in the old ANC programme was 11.1 per 1000 births (95% CI 10.4–11.8), compared to 12.2 per 1000 births (95% CI 11.4–13.1) in the new program (the difference between proportions was statistically significant *p* = 0.04).

Women who received inadequate care had 16% (AOR = 1.16, 95% CI 1.09–1.23) increased odds of delivering a newborn who was admitted to NICU and 18% increased odds of experiencing PM (AOR = 1.18, 95% CI 1.02–1.36) compared to women who received adequate care. Newborn whose mothers received intensive care had 16% increased odds of being admitted to NICU (AOR = 1.16, 95% CI 1.11–1.22) compared to newborns whose mothers received adequate care. The odds of experiencing PM among women who received intermediate care was 44% lower (AOR = 0.56, 95% CI 0.45–0.70) than for those who received adequate care (Table 4). The results we observed for the associations between APNCU index categories and NICU admission or PM in the regression models stratified by old/new ANC programme and preterm/term newborns were mainly in agreement with the results for the total study sample; women receiving inadequate care had higher odds of NICU admission (both programmes) and PM (old programme). Newborns of women receiving intensive care experienced higher odds of NICU admission (both programmes) and those with mothers receiving intermediate care had significantly lower odds of NICU admission (new programme) and PM (both programmes) compared to the adequate care group (Table 5). Term newborns of women in all APNCU index categories had increased odds of NICU admission when compared to term newborns of women receiving adequate care, while the odds of the same outcome were not statistically significant for preterm newborns. The odds of PM were lower among women receiving intensive care compared to those receiving adequate care for both term and preterm newborns; similar odds were observed for intermediate care, but only for preterm newborns. The highest odds of PM were found among term newborns whose mothers received inadequate care (Table 6).

**Table 4:** Crude and adjusted odds ratios (OR) and 95% confidence intervals (CIs) for the associations between Adequacy of Prenatal Care Utilization index categories and neonatal intensive care unit (NICU) admission and perinatal mortality (PM) among singleton newborns.

Exposure	NICU admission			PM		
APNCU index category	n cases	OR (95% CI)	AOR (95% CI)	n cases	OR (95% CI)	AOR (95% CI)
*Intensive*	3887	1.18 (1.12–1.23)	1.16 (1.11–1.22)	501	0.89 (0.80–0.99)	0.91 (0.81–1.03)
*Adequate*	3735	1.0	1.0	618	1.0	1.0
*Intermediate*	936	0.98 (0.91–1.05)	0.97 (0.90–1.05)	92	0.59 (0.48–0.72)	0.56 (0.45–0.70)
*Inadequate*	1587	1.25 (1.18–1.32)	1.16 (1.09–1.23)	284	1.45 (1.28–1.65)	1.18 (1.02–1.36)

The models are adjusted for maternal age, parity, education, region of residency and delivery.

**Table 5:** Adjusted odds ratios (AOR) and 95% confidence intervals (CI) for the associations between Adequacy of Prenatal Care Utilization index categories and neonatal intensive care unit (NICU) admission and perinatal mortality among singleton newborns for women eligible for the old and new antenatal care (ANC) programmes, separately.

	Old ANC programme	New ANC programme
**NICU admission**	AOR (95% CI)	AOR (95% CI)
Intensive	1.17 (1.09–1.25)	1.14 (1.07–1.22)
Adequate *(reference)*	1.0	1.0
Intermediate	1.07 (0.95–1.20)	0.82 (0.74–0.90)
Inadequate	1.14 (1.04–1.25)	1.10 (1.01–1.20)
**Perinatal mortality**		
Intensive	0.90 (0.77–1.06)	0.91 (0.76–1.09)
Adequate *(reference)*	1.0	1.0
Intermediate	0.63 (0.45–0.88)	0.48 (0.36–0.65)
Inadequate	1.27 (1.04–1.54)	1.06 (0.86–1.31)

The models are adjusted for maternal age, parity, education, region of residency and delivery.

**Table 6:** Adjusted odds ratios (AOR) and 95% confidence intervals (CIs) for the associations between Adequacy of Prenatal Care Utilization index categories and neonatal intensive care unit (NICU) admission and perinatal mortality among preterm and term singleton newborns.

	Preterm (GA week 23–36)	Term (GA week 37–43)
**NICU admission**	AOR (95% CI)	AOR (95% CI)
Intensive	1.00 (0.91–1.10)	1.08 (1.01–1.15)
Adequate *(reference)*	1.0	1.0
Intermediate	1.09 (0.91–1.29)	1.14 (1.04–1.25)
Inadequate	1.06 (0.93–1.20)	1.14 (1.05–1.23)
**Perinatal mortality**		
Intensive	0.72 (0.63–0.84)	0.76 (0.59–0.97)
Adequate *(reference)*	1.0	1.0
Intermediate	0.67 (0.50–0.89)	0.82 (0.57–1.19)
Inadequate	0.98 (0.82–1.17)	1.37 (1.05–1.79)

GA: gestational age.

The models are adjusted for maternal age, parity, education, region of residence and delivery.

The associations between GA at ANC initiation and NICU admission and PM were not statistically significant, except for late ANC initiation (at GA week 13–28), which increased the odds of NICU admission by 14% (AOR = 1.14, 95% CI 1.06–1.23) compared to those with early ANC initiation (before GA week 12).

## Discussion

In this study of singleton pregnancies from Georgia in 2017–2019, we found that the majority of pregnant women did not receive adequate ANC (62%) and that the utilization of ANC was significantly associated with NICU admission and PM. The lowest PM rate was found among women who received intermediate care, and the highest rate among women with inadequate care. Specifically, our results indicate that inadequate care (no ANC, less than 50% of recommended ANC visits, or ANC initiation after the first trimester) increased the odds of newborn admission to NICU by 16% and the odds of PM by 18%. These findings are in line with other studies [1, 5, 6, 26]. Another important finding was that women who received intensive (36%) and inadequate care (16%) were overrepresented in our study sample, representing higher proportions than in other countries [6, 21, 27, 28].

Previous studies have suggested a U-shaped relationship between ANC utilization and PM. Women with both inadequate care and extra ANC visits have shown higher risks of poorer birth outcomes [29–31], as extra ANC visits are determined by morbidity during pregnancy. In our study, women receiving intermediate care had 44% decreased odds of experiencing PM compared to those receiving adequate care. Women who received intensive care did not experience higher odds of PM than those receiving adequate care. On the other hand, they experienced 16% increased odds of newborn admission to NICU, which may indicate a higher proportion of morbidity in these pregnancies, although no increase in PM rate. There are several possible explanations for this finding. Theoretically, women who receive intensive care are more likely to have a high-risk pregnancy, and they could have benefited from these extra ANC visits and from NICU admission. However, the number of women receiving intensive care was larger in Georgia compared to other countries, which may suggest an overuse of medical services in this subset of pregnant women. The high proportion of women attending more than recommended number of ANC visits could be an effect of some healthy women, especially older, nulliparous women with higher education, attending self-initiated extra ANC visits that are not necessary from a medical point of view. Further, the fact that women who received intermediate care experienced the lowest odds of PM and NICU admission, may suggest that: 1) those with low-risk pregnancies do not necessarily have to complete all recommended ANC visits, as the intermediate care group seems to include women with the healthiest, morbidity-free pregnancies; and 2) that well-performed screening during ANC properly identifies high-risk pregnancies. This suggests that the number of ANC visits should be determined on an individual basis for each women, based on risk assessment. These suggestions are supported by previous research, which has shown that goal-oriented, effective ANC with four or five visits does not affect perinatal outcomes when compared to eight ANC visits, but is more cost-effective [32]. Furthermore, over-medicalization of childbirth has become common practice in many settings, which can include unnecessary, or even inappropriate interventions [33]. Limited access to data about maternal complications did not allow us to investigate the reasons for extra ANC visits, and although the proportion of women who received intensive care was higher than expected, we cannot confirm our hypothesis about unnecessary ANC visits among a subgroup of women receiving intensive care.

In line with other studies, we observed increased odds of NICU admission and PM in women receiving inadequate care [27, 34]. The odds of NICU admission for the inadequate care group, i.e., women who initiated ANC after GA week 14 or attended <50% of recommended ANC visits, increased by 16% compared to the adequate care group. Similar to our findings, previous research reported statistically significant, increased odds of NICU admission for extremely preterm newborns without active antenatal management [34], severe perinatal morbidity for all newborns [30], as well as increased risk of stillbirth and ENDs [6] and preterm birth [21, 31]for women in the inadequate care group, compared to the adequate care group.

The majority of pregnant women in Georgia who delivered singleton newborns during the study period started their ANC before GA week 12 (85%). The timing of ANC did not seem to have a substantial impact on NICU admission or PM, except among women who initiated ANC at 13–28 GA weeks, who experienced higher odds of their newborns being admitted to NICU. A similar finding was provided by a study from Cape Town, which suggested that ANC initiation was not a determinant of stillbirth [35].

Another important finding of our study was that there was no decline in the proportion of NICU admissions or PM after the implementation of the new ANC programme, which supports the idea that the number of ANC visits itself does not have an impact on the outcome, but the risk assessment during ANC, and thus, quality of care, is much more important. In fact, there was a significant increase in both NICU admissions and PM and after the implementation of the new ANC programme. Nevertheless, our rather short study period may not be long enough to draw valid conclusions. Also, the effectiveness of the new ANC programme cannot be evaluated only by the proportions or ORs for different time points; therefore more research that takes into account morbidity data is needed. However, our findings indicate that the new ANC programme does not reduce NICU admissions or PM in Georgia. Thus, it might be that the number of ANC increased with the new ANC programme, but the quality and amount of ANC provided remained the same.

Furthermore, stratified analyses by pretem/term birth showed that NICU admission was significantly associated with intensive, intermediate, and inadequate care among newborns born after completed GA week 37. Interestingly, the odds of PM were lower for term and preterm newborn in the intensive care group. These findings strengthen the above-mentioned explanations–women receiving intensive care benefited from extra ANC visits; however, this group might include women with self-initiated unnecessary visits. Additionally, this analysis showed that the impact of ANC utilization on NICU admission and PM, especially for inadequate care, is larger among women who delivered after completed GA week 37, and this finding is in line with previously reported results [36, 37].

Only a few studies have assessed the association between ANC utilization and NICU admission/PM, and this study represents the first attempt to address this issue in the Georgian population. The main strength of the study was that we included all women who delivered a singleton baby in Georgia during a 3-year period. The main outcome was validated by the VRS, and the agreement between the GBR and the VRS was high (99.8%). Additionally, we used the most recent and valid index for ANC utilization, and the number of ANC visits was calculated based on current practice and guidelines in Georgia.

The main concern in studies that assess the relationship between ANC and pregnancy outcomes is the definition of adequacy of care. Crudely, number of ANC visits, even after adjustment for GA and ANC initiation, does not precisely assess the impact of ANC on the outcomes [38]. No indices that exist for assessing ANC utilization take into account the quality of care provided during ANC visits. Therefore, neither the quality of provided care, nor the risk conditions of woman is considered in the results derived from this index, which can be seen as a limitation of this study. The APNCU index has also been criticized for introducing bias, since women shorter pregnancies and frequent ANC visits are more likely to be included in the intensive care group, which complicates the possibility of studying the impact of a ANC on preterm birth or low-birth weight newborns [39]. However, all indices have arbitrary thresholds, which always leads to slight, possible misclassifications between groups, although it does not lead to substantial bias for associations between index groups and outcomes [29]. Additionally, we did not analyse high-risk women separately, and the final analyses were not adjusted for maternal diseases during pregnancy, as complete information about maternal diseases during pregnancy is not yet available in the GBR. However, we assume that the results would only be slightly different if we could adjust for maternal diseases, as GA at delivery and maternal diseases are strongly correlated, and length of gestation is considered in the APNCU index itself. Additionally, other studies did not adjust for maternal diseases, and those who did, did not observe substantially different results after controlling for maternal diseases [6, 40].

Despite the limitations, the present results provide support for the conceptual premise that increasing the number of ANC visits does not have a significant impact on reducing NICU admissions or PM rates in a country like Georgia. All women should be offered the minimum set of ANC regardless of their risk level, and women who need additional care should be identified. As has been shown in other countries, separate definitions of routine recommended care should be created for high- and low-risk pregnancies, and additional ANC visits should be scheduled based on individual women’s needs [41, 42]. Inadequate care, as well as unnecessary overutilization of ANC, can lead to harmful outcomes for women and newborns. Our study results highlight the importance of studying maternal knowledge in reproductive health as well as investigating the quality of ANC.

## Conclusion

Women receiving inadequate care had the highest odds of NICU admission and PM, whereas women with intermediate care during pregnancy experienced the lowest odds. Sixty-two percent of pregnant women who delivered in Georgia during 2017–19 did not receive adequate care. Increasing the number of ANC visits does not seem to be effective for improving NICU admission or PM rates.
